# Structural Definition Is Important for the Propagation of the Yeast [*PSI*^+^] Prion

**DOI:** 10.1016/j.molcel.2013.05.010

**Published:** 2013-06-06

**Authors:** Ricardo Marchante, Michelle Rowe, Jo Zenthon, Mark J. Howard, Mick F. Tuite

**Affiliations:** 1 Kent Fungal Group, School of Biosciences, University of Kent, Canterbury, Kent CT2 7NJ, UK

## Abstract

Prions are propagated in *Saccharomyces cerevisiae* with remarkable efficiency, yet we know little about the structural basis of sequence variations in the prion protein that support or prohibit propagation of the prion conformation. We show that certain single-amino-acid substitutions in the prion protein Sup35 impact negatively on the maintenance of the associated prion-based [*PSI*^+^] trait by combining in vivo phenotypic analysis with solution NMR structural studies. A clear correlation is observed between mutationally induced conformational differences in one of the oligopeptide repeats (R2) in the N terminus of Sup35 and the relative ability to propagate [*PSI*^+^]. Strikingly, substitution of one of a Gly-Gly pair with highly charged residues that significantly increase structural definition of R2 lead to a severe [*PSI*^+^] propagation defect. These findings offer a molecular explanation for the dominant-negative effects of such psi-no-more (PNM) mutations and demonstrate directly the importance of localized structural definition in prion propagation.

## Highlights

• Mutations in a Gly^58^-Gly^59^ pair in Sup35 inhibit propagation of the [*PSI*^+^] prion
• Biochemistry of the amino acid introduced dictates the resulting phenotype
• Prion propagation defects correlate with local changes in Sup35 structure
• Increased local structural definition is associated with impaired prion propagation

## Graphical Abstract

**Figure:**
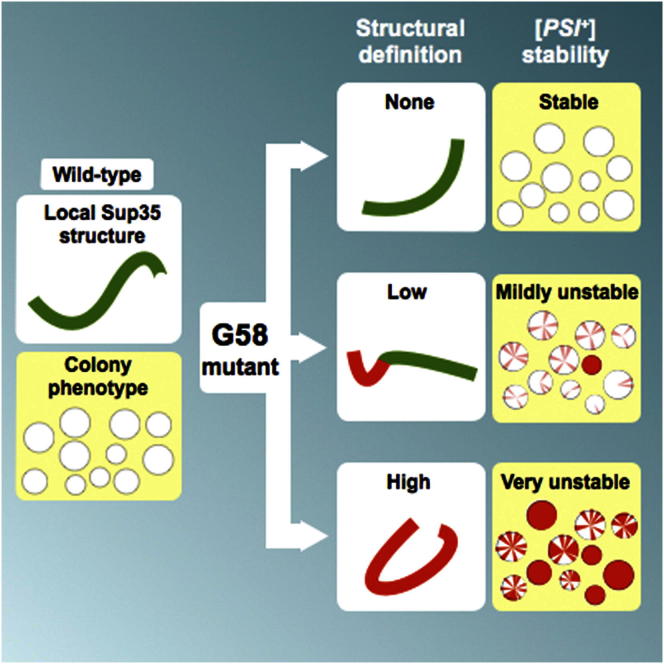

## Introduction

Prions are an important subclass of amyloid-forming proteins because of their infectious and self-propagation characteristics (Chiti and Dobson, 2006; Tuite and Serio, 2010). In mammals, the prion PrP^Sc^ is associated with a group of neuropathologies (Aguzzi and Calella, 2009), while in fungi such as *Saccharomyces cerevisiae* and *Podospora anserina*, prions act as natural epigenetic regulators of a range of different cell functions (Suzuki and Tanaka, 2013; Tuite and Serio, 2010). Uniquely, both mammalian and fungal prions can be stably propagated as one of a number of conformational variants called “strains” (in mammals) or “variants” (in fungi). Fungal prion variants have no underlying changes in the amino acid sequence of the prion protein but do have variant-specific effects on the manifestation of the prion-associated phenotype(s) (Bradley et al., 2002; Derkatch et al., 1996; King, 2001; Ohhashi et al., 2010). Yet we know remarkably little about the underlying structural basis of amino acid sequence variations in the prion protein that support or prohibit propagation of the prion variant conformations.

Fungal prion proteins have discrete regions (termed prion-forming domains, PrDs) (Tuite, 2000) that are necessary for seeded aggregation of the proteins into amyloid fibrils, their subsequent propagation in dividing cells, and the generation of specific prion variants (Bradley and Liebman, 2004; King, 2001). Yeast PrDs are enriched in Gln (Q) and Asn (N) residues (Alberti et al., 2009; Harrison et al., 2007; Michelitsch and Weissman, 2000; Sondheimer and Lindquist, 2000) that are responsible for the high propensity of these proteins to take up amyloid forms. Such diagnostic sequence features have informed the search for new prions in yeast (Sondheimer and Lindquist, 2000; Toombs et al., 2010; Alberti et al., 2009). Native PrDs in *S. cerevisiae* are typically more rich in Asn than Gln residues (Alberti et al., 2009; Toombs et al., 2010), and this most likely reflects intrinsic differences in the way Gln and Asn residues contribute to prion formation and propagation (Halfmann et al., 2011). However, the higher propensity for Pro residues to be associated with Gln-rich rather than Asn-rich regions may also be a contributory factor (Toombs et al., 2010).

**Figure 1:**
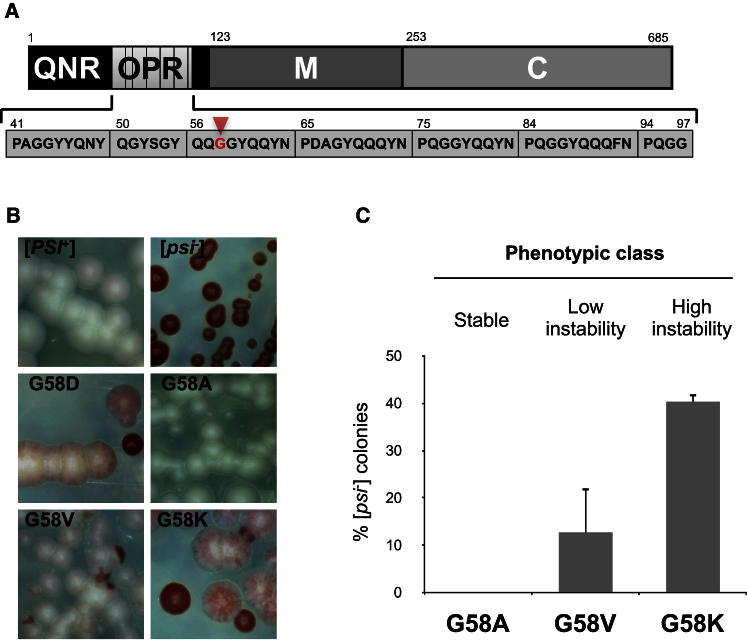
Sup35^G58X^ Mutations Result in [*PSI*^+^] Instability (A) The Sup35 protein consists, as indicated, of a C-terminal region with translation termination properties, a highly charged region (M) that is important for mitotic stability of the prion, and an N-terminal region that contains its prion forming domain (PrD; 1–97). The PrD is subdivided into the Gln/Asn-rich region (QNR) and the oligopeptide repeat region (OPR). The location of the *PNM2-1* mutation (G58D) in the second repeat of the OPR region is indicated by a red arrow. (B) Analysis of the [*PSI*^+^] instability at the single colony level caused by G58 mutations. Microscopic images of colonies (200× magnification) are shown for four different G58X mutants as indicated. The observed instability phenotypes were dependent on the residue placed at G58: highly unstable mutants (G58D, G58K) produced red [*psi*^−^] colonies and heavily sectored [*PSI*^+^] colonies, whereas low instability mutants (G58V) gave rise to loss of [*PSI*^+^] at a lower frequency and possessed a less marked sectoring phenotype. Some G58X mutations produce stable smooth white colonies, phenotypically indistinguishable from wild-type [*PSI*^+^] (e.g., G58A). (See Figure S1 for a full analysis.) (C) Levels of instability observed for three different G58X mutants representing the three different phenotypic classes as assessed by scoring the number of red colonies: no instability (G58A), low instability (G58V), and high instability (G58K). Values represent the mean ± SD for three experiments (G58K versus G58V p < 0.007). See also Figures S1–S3.

Sequence features essential for PrD function in vivo have largely emerged from studies on the yeast [*PSI*^+^] prion, an altered form of the translation termination factor Sup35 (Patino et al., 1996; Paushkin et al., 1996; Wickner, 1994). Sequestration of Sup35 into [*PSI*^+^] aggregates impairs translation termination, leading to nonsense suppression that provides a simple phenotypic assay for detecting [*PSI*^+^] in cells (Cox, 1965; Liebman and Sherman, 1979). The N-terminal PrD of Sup35 has two distinct regions that contribute to polymerization of Sup35 and propagation of [*PSI*^+^]: a Q/N-rich region (QNR; residues 1–40) and an oligopeptide repeat region (OPR; residues 41–97), respectively. The OPR is composed of five imperfect peptide repeats (consensus PQGGYQQYN; Figure 1A) that are similar in sequence to the octarepeats found in mammalian PrP (PHGGGWGQ). The Sup35-QNR region promotes the nucleation and subsequent polymerization of Sup35 (Chien and Weissman, 2001; DePace et al., 1998), with the Q and N residues most likely critical for stabilizing the amyloid state (DePace et al., 1998) and forming the amyloid core (Krishnan and Lindquist, 2005; Toyama et al., 2007; Verges et al., 2011). The Sup35-OPR region is important for the continued propagation of [*PSI*^+^] either by stabilizing Sup35-Sup35 interactions (Parham et al., 2001) and/or by providing direct binding sites for the chaperones responsible for amyloid fibril fragmentation or by altering the conformation of the amyloid core to allow chaperone access (Osherovich et al., 2004; Shkundina et al., 2006). In phenotypically “weak” [*PSI*^+^] variants, the Sup35-OPR also forms part of the amyloid core (Toyama et al., 2007; Verges et al., 2011).

To establish the critical primary sequence and/or secondary structural features for efficient prion propagation, we have analyzed Sup35 mutants unable to propagate [*PSI*^+^] (Doel et al., 1994; Young and Cox, 1971). The first such mutant described, *PNM2-1* (PNM, psi-no-more), has a single-amino-acid substitution (G58D) in the second repeat (R2) of the Sup35-OPR (Doel et al., 1994; Kochneva-Pervukhova et al., 1998). The Sup35^G58D^ mutant protein is functional and is able to enter prion aggregates but in so doing severely impairs [*PSI*^+^] propagation (Derkatch et al., 1999; DiSalvo et al., 2011; King, 2001; Kochneva-Pervukhova et al., 1998; Osherovich et al., 2004; Verges et al., 2011), resulting in loss of [*PSI*^+^] (Osherovich et al., 2004). Single-amino-acid substitutions can also affect the stability of the mammalian prion PrP^Sc^; for example, a naturally occurring human PrP polymorphism (PrP^M129V^) influences the folding and oligomerisation of the protein (Tahiri-Alaoui et al., 2004; Zeidler et al., 1997) and can prevent the development of Creutzfeldt-Jakob disease (Palmer et al., 1991). Thus, a single-amino-acid substitution in a single repeat can impair the stability and/or heritability of a yeast prion, yet little is known about the underlying molecular basis. Using a comprehensive collection of sup35 mutants, we establish the structural basis of why amino acid substitutions in the conserved Gly58Gly59Tyr60 motif in the repeat R2 of the Sup35-OPR are incompatible with efficient [*PSI*^+^] propagation.

## Results

### Not All Sup35^Gly58^ Mutations Inhibit [*PSI*^+^] Propagation

The original *PNM2-1* (Sup35^G58D^) mutant has a nonconservative substitution of Asp for the first Gly in the GlyGlyTyr motif in the second repeat of the Sup35-OPR (Doel et al., 1994) (Figure 1A). To establish whether the associated prion propagation defect was a consequence of disruption of the Gly58-Gly59 pair or due to the introduction of a hydrophilic negatively charged residue, we substituted Gly58 with a range of different amino acids. The resulting mutant *sup35* genes, expressed in single copy from their native promoter, were introduced into LJ14, a haploid [*PSI*^+^] strain carrying a *SUP35::loxP* allele covered by a plasmid-borne copy of the wild-type *SUP35* gene. The “merodiploids” so constructed all showed the original [*PSI*^+^] phenotype (data not shown), indicating that with the strong [*PSI*^+^] variant in LJ14, all the constructed *sup35* alleles were recessive. The original *PNM2-1* allele also shows recessive behavior with certain [*PSI*^+^] variants (Derkatch et al., 1999; Verges et al., 2011).

Cells expressing only the mutant Sup35^G58X^ were selected for on 5FOA-containing medium and analysis of the [*PSI*^+^] nonsense suppression phenotype showed that the Sup35^G58X^ mutants fell into three different classes: (1) no loss of [*PSI*^+^] for the Ala, Ser, Thr, Cys, Gln, Phe, Tyr, Asn, and Trp substitutions; (2) a low level of [*PSI*^+^] loss as judged by the occasional appearance of red Ade^−^ colonies for the Leu, Ile, Val, and His substitutions; and (3) a high frequency of [*PSI*^+^] loss for the Pro, Asp, Lys, Glu, and Arg substitutions (Figures 1B and 1C and Figures S1A, S1B, and S2 available online).

One Sup35^G58X^ mutant from each class was subject to further analysis, i.e., Ala (stable), Val (low instability), and Lys (high instability), together with the original Sup35^G58D^ *PNM2-1* mutant. The instability phenotype seen for both the Sup35^G58V^ and Sup35^G58K^ mutants also manifested at the individual colony level with no pure [*PSI*^+^] colonies being observed (Figure 1B). However, prion-free [*psi*^−^] sectors were observed at different frequencies depending on the substitution. No instability was observed with the Sup35^G58A^ mutant, while the original *PNM2-1* mutant (Sup35^G58D^) showed a similar high level of instability to the Sup35^G58K^ mutant.

The [*PSI*^+^] instability seen with the Sup35^G58V^ and Sup35^G58K^ mutants remained after several rounds of reculturing of sectored colonies on agar plates. The instability was quantified by determination of the frequency with which pure red [*psi*^−^] colonies were generated for a given strain (Figure 1C). The resulting [*psi*^−^] clones did not revert back to [*PSI*^+^]. Thus a range of different amino acid substitutions at Gly58 lead to a defect in [*PSI*^+^] propagation the severity of which reflected the properties of the amino acid introduced with the most severe effect being seen with hydrophilic/charged residues such as Lys and Arg.

### Sup35^G58X^ Proteins Can Form SDS-Resistant Aggregates

**Figure 2:**
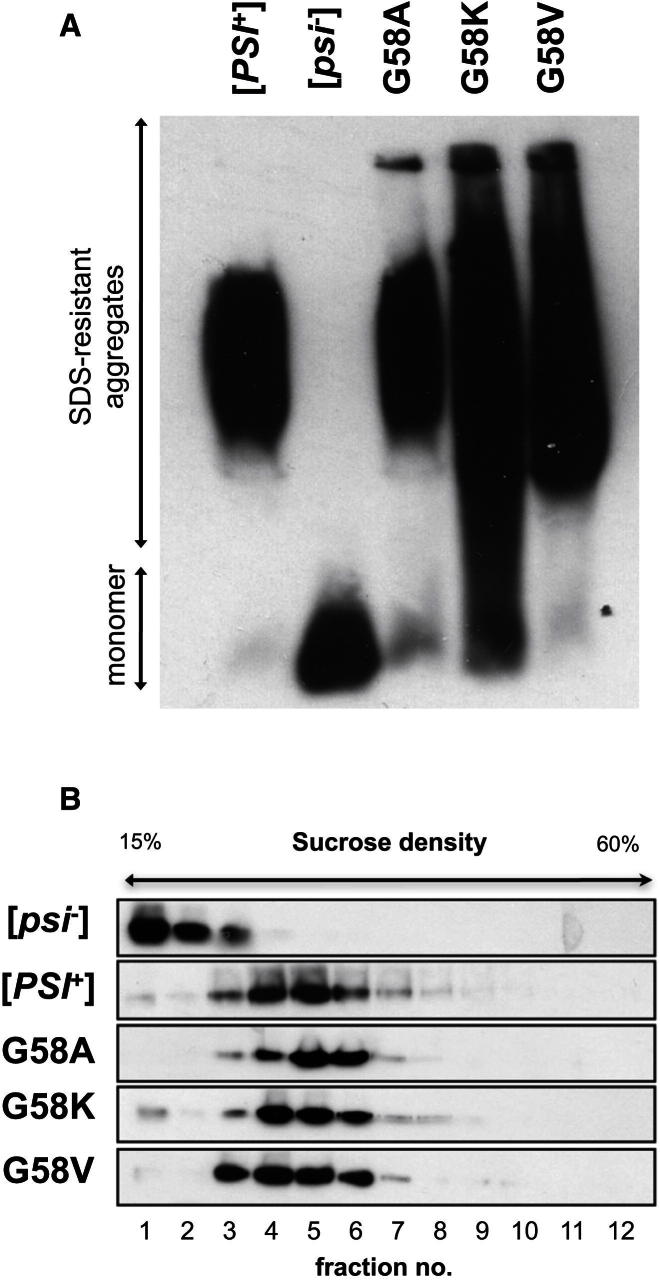
Sup35^G58^ Mutant Proteins Can Propagate High-Molecular Weight SDS-Resistant Polymers (A) SDD-AGE analysis (Kryndushkin et al., 2003) of Sup35-containing aggregates formed by the different mutant proteins. Mutants from the different instability classes are all able to form such aggregates, albeit with different polymer size distribution. (B) Native sucrose gradient analysis (15%–60%) of Sup35-containing aggregates. The average molecular mass of the aggregates did not differ significantly from wild-type to mutant Sup35.

In all [*PSI*^+^] variants so far examined, Sup35 is associated with high-molecular-weight SDS-resistant aggregates (Kryndushkin et al., 2003; Patino et al., 1996; Paushkin et al., 1996). To determine whether the [*PSI*^+^] instability seen with Sup35^G58X^ mutants reflected a failure to form such aggregates, we analyzed the mutants using semidenaturing detergent agarose gel electrophoresis (SDD-AGE) (Kryndushkin et al., 2003). All Sup35^G58X^ mutants analyzed showed similar steady-state levels of Sup35 (Figures S3A and S3B). The stable Sup35^G58A^ mutant formed SDS-resistant aggregates similar to the control [*PSI*^+^] strain, whereas the SDS-resistant aggregates formed by the Sup35^G58K^ and Sup35^G58V^ proteins showed a more disperse range of sizes, although a significant proportion of these aggregates were similar in size to the wild-type aggregates (Figure 2A).

Analysis of the Sup35-containing aggregates under native conditions with sucrose density gradients (Figure 2B) indicated only minimal differences in the native molecular weight of the mutant protein aggregates. The low amount of Sup35 routinely seen in fraction 1 for the Sup35^G58K^ mutant most likely reflects the existence of [*psi*^−^] cells in the population (see Figure 1C). Thus, the Sup35^G58X^ mutant proteins examined were still able to form protein aggregates in vivo in the absence of wild-type Sup35, albeit with some subtle changes in supramolecular organization.

### Impact of the Sup35^G58X^ Mutations on the Structure of Oligopeptide Repeat 2

As conformational flexibility is an important functional property of a PrD, we next assessed the impact the various Sup35^G58X^ mutations had on the structure of the Sup35 molecule using nuclear magnetic resonance (NMR) analysis of 13-mer peptides encompassing R2 of the Sup35-OPR (see the Supplemental Experimental Procedures). Proteins rich in Gln and Asn residues are highly insoluble and difficult to study by conventional NMR and X-ray crystallographic methods. Attempts by ourselves (unpublished data) and others (e.g., King et al., 1997) to analyze the structure of the full-length Sup35-PrD by NMR were hindered by the propensity of the protein to form insoluble amyloid aggregates. Importantly, R2 lies outside the tightly packed core of β sheets that are diagnostic of strong variants of [*PSI*^+^] (Toyama et al., 2007).

**Figure 3:**
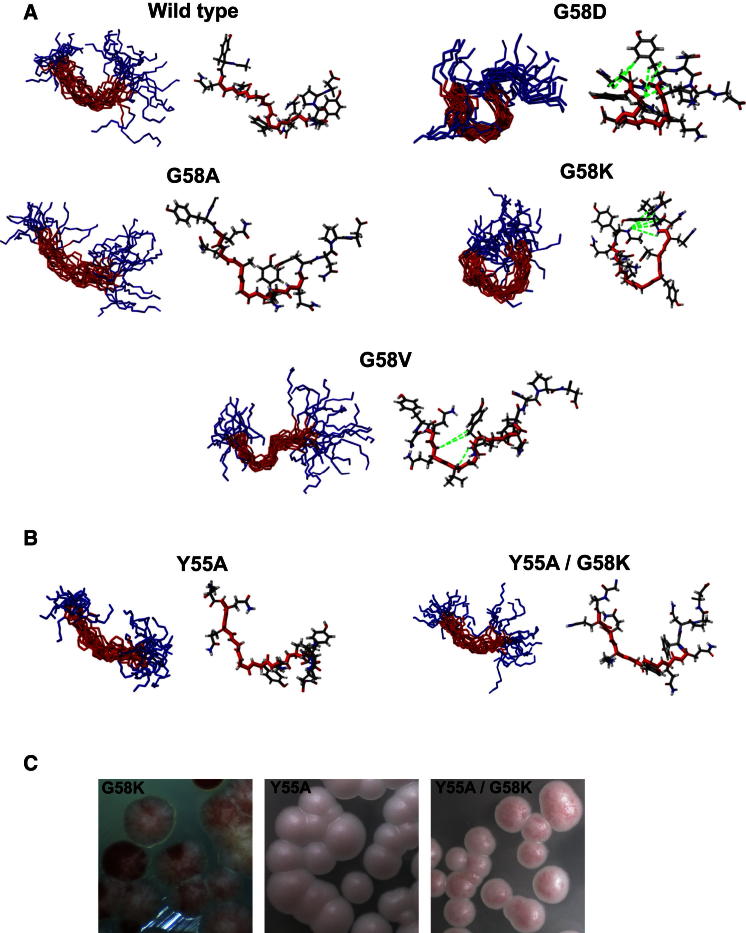
[*PSI*^+^] Instability Caused by G58 Mutations Correlates with Structural Constraints Imposed by the Mutations (A) Structures of 13-mer peptides (^54^GYQQXGYQQYNPD^66^) containing the second repeat (R2) of Sup35 OPR region determined by NMR analysis. The wild-type and four different G58 mutants are shown. The wild-type and G58A peptides exhibit an open structure, while mutants causing high [*PSI*^+^] instability (G58D, G58K) display a horseshoe-like structure. The low-instability mutant (G58V) has an intermediate S-like structure. Ensemble structures (left) and the structure closest to the mean calculated (right) are shown with intramolecular contacts in the G58D, G58K, and G58V peptides highlighted in green. (B) Structure of 13-mer peptides carrying either the Y55A or Y55A/G58K substitutions. The Y55 substitution suppresses the structural definition caused by the G58K mutation. (C) Phenotypic analysis of G58K, Y55A, and Y55A/G58K double mutants. The Y55A mutation significantly reduces [*PSI*^+^] instability caused by the G58K mutation. See also Figure S4 and Table S1.

The structures of various R2 peptides containing one or other of the representative Sup35^G58X^ substitutions were solved by NMR (Figure 3A). The wild-type R2 peptide displayed an extended conformation, as did the R2^G58A^ peptide with no long-range interresidue contacts. In contrast, the R2^G58D^ and R2^G58K^ peptides adopted a curved, “horseshoe”-like conformation supported by long-range contacts between distant residues (for NOE/ROE contacts, see Figure S4). The R2^G58D^ peptide showed contacts between Gln56, Gln57, and Tyr63, while R2^G58K^ displayed contacts between Tyr55, Tyr63, and Pro65. These contacts defined the curved structures observed that were composed of 7 and 8 amino acids, respectively. R2^G58V^ was unique in showing only long-range contacts between residues 3 amino acids apart (i.e., Gln57-Tyr60) that resulted in a tight bend in this region with no further structure definition for the rest of the peptide.

Two additional mutants, Sup35^Y55A^ and Sup35^Y55A/G58K^, verified the role of tyrosine residues in supporting the formation of curved structures (Figures 3B and 3C). The Sup35^Y55A/G58K^ mutation almost completely abolished the [*PSI*^+^] instability caused by G58K with only a low level of instability at the colony level being observed (Figure 3C) with [*psi*^−^] colonies only representing 0.25% ± 0.2% of the total compared with 40% ± 2% seen for the Sup35^G58K^ mutant. Taken together, these data suggest pi-pi stacking of the Tyr55 stabilized the curved structural arrangements in R2 and the structural constraints caused by the G58K mutant are the primary driver of the observed [*PSI*^+^] instability associated with this mutant.

Importantly, these data establish a clear correspondence between extended structure and high prion stability (wild-type, Sup35^G58A^) and between curved structure and high prion instability (Sup35^G58D^, Sup35^G58K^), with the intermediate “bend-extended” structure (Sup35^G58V^) corresponding to the intermediate prion stability phenotype. These findings thus provide a credible physical reason for the different effects of the various G58 substitutions.

### Expanding the Phenotypic Analysis to Gly59 and Tyr60

**Figure 4:**
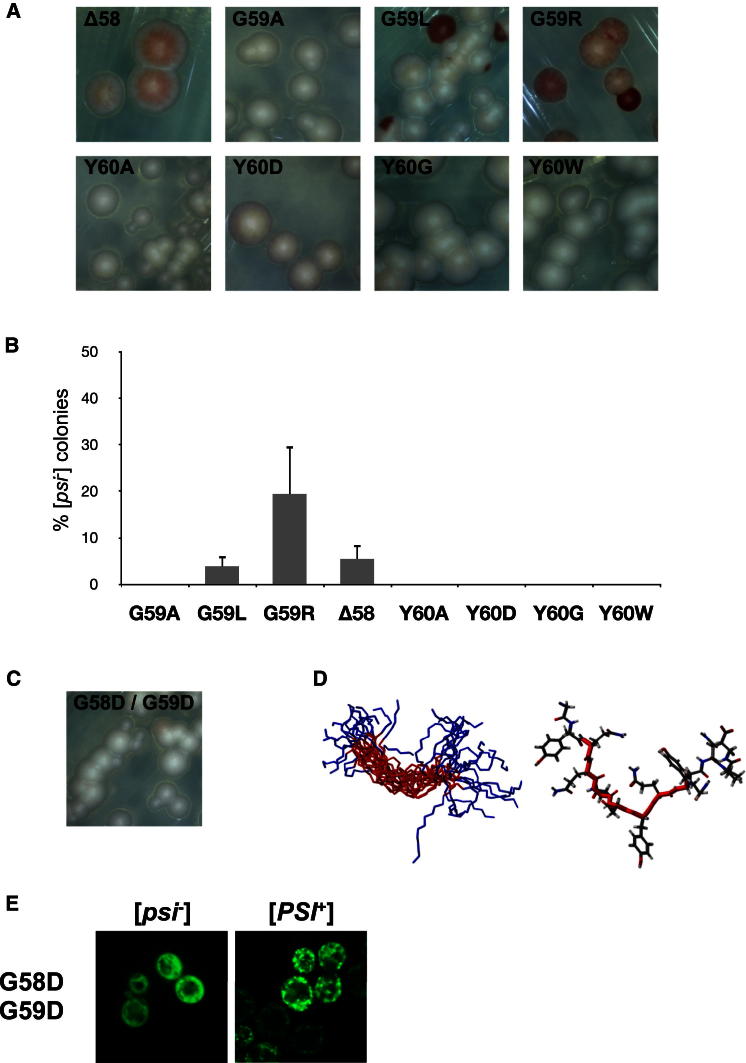
Phenotypic Analysis of Δ58, G59, and Y60 Mutants (A) Colonies of cells expressing Sup35^G59X^ mutant proteins show all the same [*PSI*^+^] instability observed with Sup35^G58X^ mutants i.e., no instability (G59A), low instability (G59L), and high instability (G59R). Mutations at Y60 do not inhibit [*PSI*^+^] propagation, while deletion of Gly58 (Δ58) results in a very low level of instability. (B) Levels of [*PSI*^+^] instability of the Sup35^G59X^ mutants shown in (A). Values represent the mean + SD for three experiments (G59L versus Δ58, p < 0.060; G59R versus Δ58, p < 0.082). (C) The colony-level phenotype of the Sup35^G58D/G59D^ double mutant is phenotypically indistinguishable from that of the wild-type. (D) NMR structure of a 13-mer peptide with the G58D/G59D mutation (peptide sequence ^54^GYQQDDYQQYNPD^66^) shows that this mutant peptide lacks structural definition similarly to the wild-type. (E) Overexpression of Sup35NM-GFP^G58D/G59D^ in both [*psi*^−^] and [*PSI*^+^] derivatives of 74D-694. Punctate and diffuse fluorescence patterns characteristic of [*PSI*^+^] and [*psi*^−^] states, respectively, could be observed after 4 hr overexpression, showing that this mutant can decorate wild-type prion aggregates when overexpressed. See also Figure S4 and Table S1.

The Gly-Gly pair in R2 of Sup35-OPR would be expected to confer a high degree of conformational flexibility to this region (Gazit, 2002b), and therefore mutation of Gly59 would be expected to result in a similar prion destabilizing effect to the Gly58 mutations. Gly59 was therefore replaced by amino acids that triggered high, low, or no instability when used to replace Gly58, namely, Arg, Leu, and Ala, respectively (Figures 4A and 4B). As with Gly58, substitution of Gly59 with either Arg or Leu resulted in [*PSI*^+^] instability, whereas the Ala substitution did not. Deletion of the Gly58 residue (Δ58) also resulted in a low level of [*PSI*^+^] instability (Figures 4A and 4B). These data confirm the importance of the Gly-Gly pair for ensuring conformational flexibility within R2 and that the nature of the amino acid side chains contributes to defining the severity of their impact on prion propagation.

Since introduction of a charged residue at either Gly58 or Gly59 affected stable [*PSI*^+^] propagation, we next examined the consequence of mutating both Gly58 and Gly59 to Asp to see whether this exacerbated the prion instability phenotype of the single mutants. Surprisingly, the Sup35^G58D/G59D^ mutant behaved like the wild-type protein, showing no prion propagation defect (Figure 4C), and the R2^G58D/G59D^ peptide (Figure 4D) had a similar extended conformation to that of the wild-type R2 peptide and R2 mutants that did not affect prion propagation. This mutant was also capable of decorating wild-type aggregates when overexpressed as a GFP fusion in a wild-type [*PSI*^+^] background (Figure 4E).

The Gly-Gly pair in R2 is followed by a Tyr residue that is conserved in the other four repeats (Figure 1A). However, replacement of the aromatic side chain of Tyr with different functional groups had no effect on the ability to stably propagate [*PSI*^+^] (Figures 4A and 4B), as was also the case for Tyr55 (Figures 3B and 3C). The residues used contained a side chain with a CH_3_ group (Ala), a polar negatively charged group (Asp), a hydrogen atom (Gly), or a different aromatic side-chain (Trp). Therefore, the conserved Tyr residue in the Gly-Gly-Tyr motif is not required for the efficient propagation of the [*PSI*^+^] prion.

### Disruption of Gly-Gly Pairs in Other Repeats Does Not Inhibit [*PSI*^+^] Propagation

**Figure 5:**
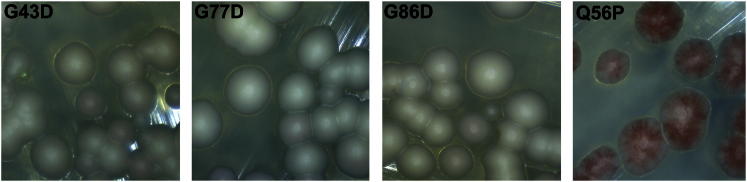
Point Mutations in Different Sup35 Repeats G to D mutations were created at the first G residue of the GGY motifs in repeats 1, 4, and 5 (G43, G77, and G86, respectively). Contrary to the *PNM* effect observed with the repeat 2 mutation (G58D), none of the mutations mentioned above had an effect of [*PSI*^*+*^] stability. Conversely, the Q56P mutation, which mutates the first Q residue in repeat 2 to the canonical P residue found at the beginning of every other repeat, caused [*PSI*^*+*^] instability. Microscopic images of colonies (200× magnification) are shown for mutants as indicated. See also Figure S5.

If the oligopeptide repeats in the Sup35-OPR define a repeating structure in the prion form of Sup35 and the instability we observe with the Sup35^G58X^ and Sup35^G59X^ mutations was due to disruption of that structure, then one might predict that introducing analogous Gly substitutions in each of the other four repeats would likewise destabilize [*PSI*^+^]. Consequently, we constructed the equivalent Sup35^G58D^ substitution in R1 (Sup35^G43D^), R4 (Sup35^G77D^), and R5 (Sup35^G86D^), and in each case [*PSI*^+^] propagation was unaffected (Figures 5 and S5). R3 has AGY instead of the GGY motif and was not analyzed. These data suggest that only R2 plays an important role in the maintenance of the [*PSI*^+^] prion state of Sup35. Interestingly, R2 is also the only repeat that starts with a Gln rather than a Pro residue (Figure 1A), and this sequence feature is important because [*PSI*^+^] instability was observed in a Sup35^Q56P^ mutant (Figures 5 and S5).

### Impact of Sup35^G58X^ Mutations on De Novo Prion Formation and Prion-Associated Toxicity

**Figure 6:**
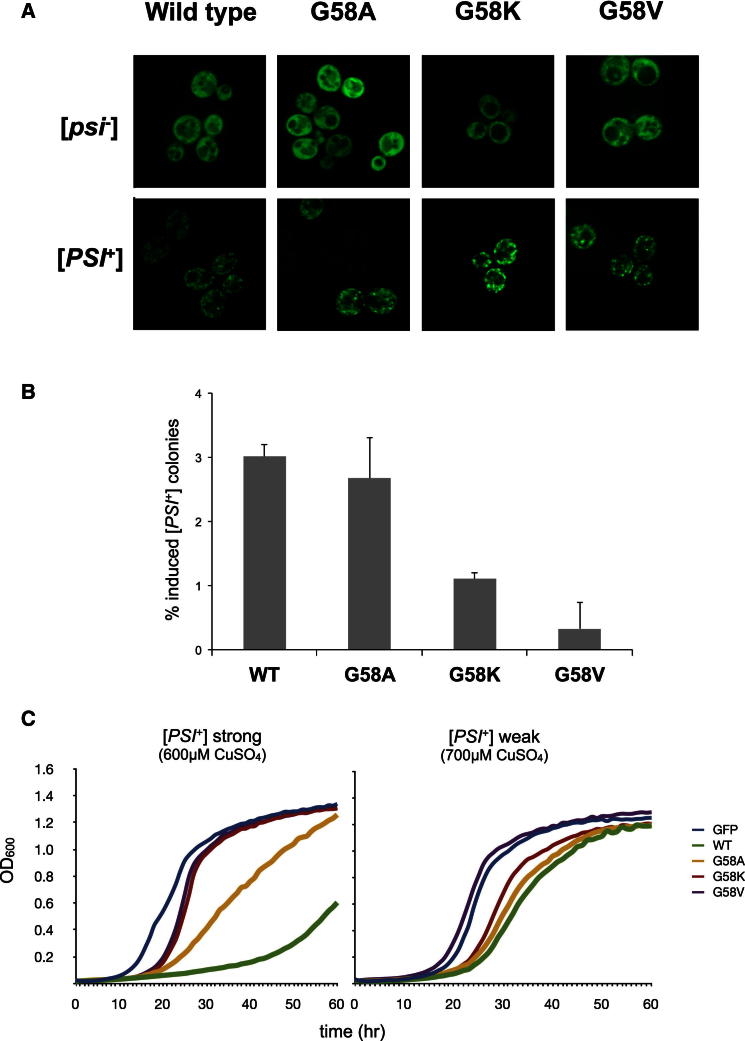
Sup35^G58X^ Mutants Decorate Wild-Type [*PSI*^+^] Aggregates but Differ in Their Ability to Induce [*PSI*^+^] De Novo and to Induce Cell Toxicity when Overexpressed in [*PSI*^+^] Background (A) Overexpression of Sup35NM-GFP and mutant derivatives in both [*psi*^−^] and [*PSI*^+^] derivatives of 74D-694, 4 hr after induction. In [*PSI*^+^] cells, both wild-type and mutant Sup35NM-GFP join pre-existing Sup35 aggregates, giving the cells the punctate fluorescence pattern characteristic of [*PSI*^+^] cells. In [*psi*^−^] cells, the Sup35-NM GFP signal is diffuse for all derivatives. (B) Sup35^G58X^ mutants differ in their ability to induce [*PSI*^+^] de novo when overexpressed. Wild-type and Sup35^G58A^ induce [*PSI*^+^] at similar frequencies, while Sup35^G58K^ and Sup35^G58V^ both show significantly lower frequencies. Values represent the mean ± SD for three experiments (WT versus G58K, p < 0.003; WT versus G58V, p < 0.017). (C) Toxicity of Sup35 and its Sup35^G58X^ mutant derivatives when overexpressed in a strain with either a weak or a strong [*PSI*^+^] variant. Different levels of toxicity were observed, with the wild-type being highly toxic to the strong [*PSI*^+^] variant and the Sup35^G58A^ mutant showing only a slight reduction in this toxicity, while Sup35^G58K^ and Sup35^G58V^ showed no toxicity at the CuSO_4_ concentrations used. Toxicity was much reduced in the weak [*PSI*^+^] variant strain even at higher concentrations of CuSO_4_. See also Figure S6.

To establish whether the mutant Sup35 proteins could decorate pre-existing Sup35 prion aggregates, we expressed three different Sup35^G58X^NM-GFP fusions in both a strong [*PSI*^+^] variant strain and a [*psi*^−^] strain. All three mutant Sup35NM-GFP proteins decorated pre-existing wild-type [*PSI*^+^] aggregates (Figures 6A and S6A), confirming that the Sup35^G58X^ proteins can be seeded by the prion form of wild-type Sup35 to form characteristic prion aggregates in vivo.

The ability of these Sup35^G58X^-NM mutants to induce the de novo formation of [*PSI*^+^] when overexpressed was also assessed. [*PSI*^+^] de novo formation relies on the presence of a second prion in the cells, [*PIN*^+^], the prion form of Rnq1 protein (Derkatch et al., 2001; Derkatch et al., 1997; Osherovich et al., 2004). Using a [*PIN*^+^] variant that gives a high frequency of [*PSI*^+^] induction (Bradley et al., 2002), we assessed the efficiency of each mutant Sup35 to induce [*PSI*^+^] in a [*psi*^−^] [*PIN*^+^] strain. Four hours after induction, the various Sup35NM^G58X^-GFP proteins formed ring-like structures characteristic of [*PSI*^+^] de novo formation (Tyedmers et al., 2010; Zhou et al., 2001) (Figure S6B). Furthermore, all three Sup35^G58X^NM-GFP mutant proteins could induce [*PSI*^+^] when overexpressed for 24 hr (Figure 6B). However, while Sup35^G58A^ induced [*PSI*^+^] at a frequency similar to that of the wild-type, the Sup35^G58K^ and Sup35^G58V^ mutants induced [*PSI*^+^] at significantly lower frequencies, although this could also reflect a high rate of prion loss. Therefore, the same G58 mutational changes that negatively impact on [*PSI*^+^] propagation also had a reduced ability to form [*PSI*^+^] de novo.

A further property of the Sup35^G58X^ mutants examined was their relative toxicity when overexpressed in strains carrying either a weak or a strong variant of [*PSI*^+^]. These variants arise as a result of different amyloid folds assumed by Sup35 in its prion form and manifest themselves phenotypically by changes in nonsense suppression efficiency, i.e., weak suppression or strong suppression. Overexpression of Sup35NM is toxic in [*PSI*^+^] cells particularly in strong [*PSI*^+^] variants compared with weak [*PSI*^+^] variants (Vishveshwara et al., 2009). This is due to the aggregation of the overexpressed Sup35NM protein and subsequent recruitment of wild-type Sup35 protein into nonfunctional prion aggregates, resulting in cell death (Vishveshwara et al., 2009). We confirmed that toxicity of the wild-type Sup35NM was highest in a strong [*PSI*^+^] variant, with Sup35^G58A^ showing similar levels of toxicity (Figures 6C and S6C). Sup35^G58K^ elicited comparably lower toxicity and then only with the weak [*PSI*^+^] variant, while Sup35^G58V^ showed no toxicity with either [*PSI*^+^] variant. These data suggest that although all of the Sup35NM mutants examined were able to interact with wild-type Sup35 and trigger its aggregation, their ability to do so was modulated by the identity of residue 58 in R2.

## Discussion

Through a combination of phenotypic analysis of a set of sup35 mutants and establishment of the impact of various amino acid substitutions on local secondary structure, we have established a clear causal link between protein structure definition in a single repeat (R2) of the Sup35-OPR and effective propagation of the [*PSI*^+^] prion. The observed imposition of conformational constraints on the N-terminal region of Sup35 also impacts on other properties of Sup35, including de novo prion generation and prion-associated toxicity.

### The Molecular Basis of *PNM* Mutants

The importance of the primary amino acid sequence of the Sup35-OPR for effective prion propagation was previously demonstrated by the dominant negative *PNM2-1* mutant (Sup35^G58D^) (DePace et al., 1998; DiSalvo et al., 2011; Doel et al., 1994; Osherovich et al., 2004; Verges et al., 2011). Two recent studies of this mutant have suggested different but plausible mechanisms for the loss of [*PSI*^+^]. One explanation is that the Sup35^G58D^ mutation destabilizes mixed Sup35:Sup35^G58D^ aggregates, leading to a defect in propagon generation (but not transmission) and enhanced Hsp104-mediated aggregate disassembly (DiSalvo et al., 2011). However, Verges et al. (2011) showed that the Sup35^G58D^ mutation disrupted the propagation of strong but not weak [*PSI*^+^] variants, an observation that we (R. Marchante and M.F. Tuite, unpublished data) and others (Derkatch et al., 1999) have also made. In this case, the loss of [*PSI*^+^] was attributed to an observed defect in propagon transmission from mother to daughter cells. This study also reported that the Sup35^G58D^ mutation did not alter the functional interaction with Hsp104, an observation that contradicted the findings of DiSalvo et al. (2011). The different effects of Sup35^G58D^ reported by these two studies most likely arise from the use of different [*PSI*^+^] variants and this highlights the complexity of the mechanism underlying the Sup35^G58D^ -induced loss of [*PSI*^+^]. The results we report here demonstrate that the imposition of highly localized structural changes by the *PNM2-1* mutation can significantly affect the ability of cells to propagate [*PSI*^+^].

### The Importance of the Repeat R2 Gly-Gly-Tyr Motif in [*PSI*^+^] Propagation

The Sup35^G58D^ mutation is located in a GlyGlyTyr motif found in four of the five repeats in the Sup35-OPR. Gly-Gly pairs with an adjacent aromatic residue are a common feature of proteins that are prone to aggregate and/or form amyloids (Gazit, 2002a). These include the mammalian prion protein PrP, which contains a GlyGlyTrp motif in its octarepeats, and another yeast prion protein, Rnq1 (Sondheimer and Lindquist, 2000). Glycine residues act as helix breakers and may facilitate the α helix to β sheet transition that is crucial to amyloid formation. Critically, Gly-Gly pairs can promote the conformational flexibility necessary to drive prion formation, as illustrated by mutations in one of the Gly-Gly pair in mammalian PrP octarepeats inhibiting PrP^Sc^ generation in vitro (Harrison et al., 2010).

Disruption of the Gly-Gly pair in repeat R2 clearly disrupts [*PSI*^+^] propagation, but the severity of this is highly dependent on the type of residue introduced and this in turn reflects the structural constraints imposed by the introduced residue. Charged amino acids (e.g., Lys, Asp) caused the most severe propagation defect and also imposed the highest level of structural definition. However, hydrophobic amino acids (e.g., Val) caused less prion instability that correlated with a lower level of structural definition, while amino acids compatible with stable [*PSI*^+^] (e.g., Ala) caused no discernable structural definition. That deletion of one of the Gly-Gly pair in repeat 2 only resulted in a mild prion instability phenotype suggests that the observed instabilities were due to charged or hydrophobic side chains rather than absence of the Gly residue per se.

Further support for the importance of a lack of structural definition in the Sup35-PrD in [*PSI*^+^] propagation came from the analysis of two other types of Sup35 mutant. In the first, substitution of the Tyr residue (Y55) residue with Ala not only abolished the defined structure observed for the G58K peptide (Figure 3B) but also almost completely eliminated the [*PSI*^+^] instability phenotype (Figure 3C). Second, a double mutant Sup35^G58D/G59D^ that we predicted to be highly detrimental to prion propagation had no effect on the structure of the peptide (Figure 4D) or [*PSI*^+^] stability (Figure 4C). This was most likely caused by a neutralization of the local environment through a sharing of charges by the two adjacent Asp residues, leading to the formation of an unstructured wild-type-like conformation.

Why is repeat R2 of the Sup35-OPR particularly important for the maintenance of [*PSI*^+^]? That the in vitro and in vivo studies all confirm that the Sup35^G58D^ mutant can still polymerize would point to fragmentation of Sup35 amyloids by the Hsp104-Hsp70-Hsp40 chaperone machinery as the critical issue. Certainly the R2 repeat is the only repeat that does not start with a Pro residue and introduction of such a residue at the start of repeat 2 does trigger [*PSI*^+^] instability (Figure 5). Pro residues are known to prevent aggregation of regions of proteins prone to aggregation i.e., to act as “gatekeepers.” This in turn can influence chaperone recognition of that region (Reumers et al., 2009). Disruption of this region by the introduction of local structural definition may also have the same effect on chaperone-mediated fragmentation.

### Structural Definition, De Novo Prion Formation, and the Species Barrier

A lack of structural definition in the R2 repeat is also important for de novo formation of [*PSI*^+^]. De novo formation of [*PSI*^+^] is mediated by interaction between Sup35 and the [*PIN*^+^] prion that may trigger its polymerization (Derkatch et al., 2004). In vitro studies have shown that the Sup35^G58D^ mutant is able to form amyloid polymers in vitro (Osherovich et al., 2004), albeit being poor at forming fibrils that can nevertheless generate and sustain the propagation of weak [*PSI*^+^] variants (Verges et al., 2011). When overexpressed in a [*PIN*^+^] strain, the two Sup35^G58X^ mutants examined that were associated with [*PSI*^+^] instability also showed a reduced ability to form the [*PSI*^+^] prion de novo (Figure 6B), although this reduced ability may also reflect a higher rate of loss once formed. The assembly of proteins such as Sup35 into amyloid is a templated process and constraining the structure of an inherently unstructured region might be expected to slow down this process, while conformational adaptation of the wild-type protein to the newly formed mutant prion would occur in a manner analogous to heterotypic prion protein interactions in the same cell (Afanasieva et al., 2011; Chen et al., 2010; Chen et al., 2007). Alternatively, there might be incompatibility between the overexpressed mutant protein and the [*PIN*^+^] variant templating the de novo formation of [*PSI*^+^], and further studies will be required to distinguish between these possibilities.

Different prion protein structures underlie the basis of prion variants, variant compatibility, and establishment of species barriers (Caughey et al., 1998; Collinge and Clarke, 2007; Derkatch et al., 2000; Derkatch et al., 1996). These, coupled with our own findings, suggest that single-amino-acid changes can have a significant impact on the local structure of the protein and change their aggregation patterns and their ability to stably maintain the [*PRION*^+^] state. The most widely discussed model for Sup35 amyloids postulates that amino acid residues interact with identical residues in neighboring molecules forming a stack of parallel in-register β sheets involving the Sup35-PrD (Ross et al., 2004, 2005), a prediction that is borne out by the observation that scrambling the Sup35-PrD sequence does not affect the ability to form the [*PSI*^+^] state per se (Ross et al., 2004; Toombs et al., 2010). However, possible changes in intramolecular interactions occurring when shuffling a prion domain’s primary sequence should be taken into consideration when looking at the propagation of such amyloids. Residue shuffling could confer structural definition to regions where such is detrimental to prion propagation or possibly disrupt chaperone binding sites. That mutation of the Gly-Gly pair in Sup35-OPR repeat 2 causes a propagation defect while the same mutation in repeats 1, 4, and 5 does not (Figure 5) suggests that repeat 2 might be one such chaperone binding site.

The importance of conformational flexibility for propagation of fungal prions has been inferred from in vitro studies (Pierce et al., 2005; Scheibel and Lindquist, 2001), and our study now provides the first strong in vivo support for this hypothesis. Studies with the mammalian PrP^Sc^ prion have also demonstrated the impact of local alterations in structural definition on prion behavior in vertebrates. For example, a natural double amino acid polymorphism (S170N, N174T) shifts the unstructured β2-α2 loop of PrP (residues 165–175) to a rigid loop structure (Gossert et al., 2005). The resulting change in local protein conformation is sufficient to generate a transmission (i.e., species) barrier between itself and wild-type PrP without the rigid loop structure (Sigurdson et al., 2009, 2010). This might reflect the generation of a variant of the PrP^Sc^ prion by the PrP polymorphism or reduction of the conformational flexibility necessary to generate a spectrum of prion variants. Previous studies have also revealed a range of interactions between Sup35 mutants and [*PSI*^+^] variants, including a limited compatibility of the Sup35^G58D^ mutant with some [*PSI*^+^] variants (Derkatch et al., 1999; Verges et al., 2011) and compatibility of certain [*PSI*^+^] variants induced by Sup35^G58D^ overexpression with both wild-type and mutant Sup35 (King, 2001).

## Experimental Procedures

### Plasmid Construction

Plasmid pUKC1620 was constructed by PCR amplification of the *SUP35* promoter (−949 to −49) with XhoI and BamHI ends and was cloned into pRS313 (Amp^r^, CEN, *HIS3*). The *SUP35* coding sequence was cloned into this plasmid as a BamHI-XbaI fragment. Mutant *SUP35* alleles were created with the QuikChange mutagenesis kit (Stratagene) using pUKC1620 as a template and the primers listed in Table S2. Site-directed mutagenesis of plasmid p6442 (*CUP1*, *SUP35*NM-GFP), kindly donated by Susan Lindquist (Whitehead Institute, Boston), was also performed with the QuikChange mutagenesis kit. Control plasmid p6431 (*CUP1*, GFP) was also donated by Susan Lindquist. All plasmids generated (Table S3) were confirmed via sequencing.

### Yeast Strains

*S. cerevisiae* strain LJ14 (*MATa ade1-14 trp1-289 his3Δ*-200 *ura3-52 leu2-3,112 SUP35::loxP* p[*SUP35-URA3*][*PSI*^+^]) was used for all shuffling experiments and phenotypic assays. This strain is a derivative of strain 74D-694 (*MATα ade1-14 trp1-289 his3Δ*-200 *ura3-52 leu2-3,112*), which was used as a control for all experiments. The [*psi*^−^][*PIN*^+^] 74D-694 strain containing a characterized variant of the [*PIN*^+^] prion was kindly donated by Susan Liebman (University of Illinois, Chicago).

### Plasmid Shuffling and Phenotypic Analysis

Plasmids were transformed into strain LJ14 with standard PEG/LiAc/ssDNA transformation and were selected for on histidine- and uracil-deficient synthetic medium. Phenotypes were scored by growth of colonies overnight at 30°C in yeast extract peptone dextrose (YEPD) medium and spotting of different dilutions of these cultures on rich ¼ YEPD agar and rich ¼ YEPD agar with 3 mM guanidine hydrochloride to confirm their [*PSI*^+^] status. Plasmid shuffling was performed by growth of double-transformed LJ14 overnight at 30°C in synthetic -His -Ura medium and then plating on YEPD supplemented with 1 mg/ml of 5-fluoroorotic acid. Phenotypes of the postshuffled colonies were assessed as described above. Magnified images of single colonies were acquired with a USB portable microscope (200× magnification). [*PSI*^+^] instability was quantified from three independent logarithmic phase cultures of the selected mutants in YEPD; cultures were diluted and plated into two ¼ YEPD agar plates (∼500 cells/plate), and the [*PSI*^+^] state of resulting colonies was scored after 4 days growth. More than 1,000 colonies were scored for each experiment, and the statistical significance was determined with a Student’s t test.

### Western Blot Analysis

Cell extracts were prepared from logarithmic phase cultures as described (von der Haar, 2007). Extracts were analyzed with a 4%–20% polyacrylamide Tris-Glycine gradient gel (Invitrogen), run at 125 V. Protein was transferred onto a polyvinylidene difluoride (PVDF) membrane by semidry blotting (10 V, 45 min), and membranes were probed with anti-Sup35 (MT50) polyclonal antibody, anti-GFP monoclonal antibody (Roche), or anti-Pgk1 polyclonal antibody (York Biosciences). Anti-rabbit and/or anti-mouse HRP-conjugated antibody was used as a secondary antibody in standard ECL analysis. For densitometry analysis, the image analysis software ImageJ (http://rsbweb.nih.gov/ij/) was used. SDS-PAGE gels loaded with the same amount of total protein and stained with Coomassie brilliant blue were used as loading controls.

### SDD-AGE Analysis

SDD-AGE analysis was performed as previously described (Kryndushkin et al., 2003) with the following modifications: cell extracts were obtained from logarithmic phase cultures of selected mutants by mechanical lysis (lysis buffer: 25 mM Tris-HCl [pH7.5], 50 mM KCl, 10 mM MgCl_2_, 1 mM EDTA, and 1 protease inhibitor cocktail tablet [Roche Applied Sciences]). Protein extracts were loaded in a 1.5% agarose gel prepared in buffer G (20 mM Tris, 200 mM glycine) and ran on Laemmli buffer (20 mM Tris, 200 mM glycine, and 0.1% SDS). Protein was transferred using semidry blotting and transfer buffer T (20 mM Tris, 200 mM Glycine, 0.1% SDS, and 15% [v/v] methanol) onto a PVDF membrane for 90 min at 10 V. MT50 anti-Sup35 antibody was used as described above.

### Sucrose Density Gradient Analysis

Sucrose gradients (15% to 60%) were freshly prepared in lysis buffer (100 mM NaCl and 10 mM EDTA in PBS with a protease inhibitor cocktail). The PBS was obtained from Sigma (P4117). Protein extracts were prepared as described for SDD-AGE and diluted to 2.5 mg/ml total protein concentration. Samples (100 μl) were pipetted onto the top of a gradient and centrifuged at 31,000 rpm, 4°C, for 3 hr. Gradients were sampled while on ice by pipetting of 160 μl fractions from the top of the gradient into prechilled tubes that were snap-frozen in liquid nitrogen and stored at −80°C for subsequent western blot analysis. Each gradient produced 13 fractions.

### Cell Imaging

The plasmid p6442 and mutated derivatives (see Table S3) were individually transformed into the yeast strain 74D-694 [*PSI*^+^]. Overexpression of the different Sup35NM^G58X^-GFP mutants was induced by addition of a final concentration of 25 μM CuSO_4_ to logarithmic growth phase cultures in SD-Ura medium. Cells were typically observed 4 hr after induction with an Olympus IX-81 fluorescence microscope with a 150 W xenon-mercury lamp and an Olympus 60× Plan NeoFluor oil-immersion objective. Images captured were deconvolved with Huygens Deconvolution Software (Scientific Volume Imaging).

### [*PSI*^+^] De Novo Induction

Overexpression of mutant Sup35NM^G58X^-GFP was induced with 50 μM CuSO_4_. Cells were harvested 24 hr after induction and were plated at an appropriate dilution onto ¼ YEPD plates that were then incubated for 4 days at 30°C. For each strain, over 1,000 colonies in three independent experiments were scored as [*PSI*^+^] or [*psi*^−^] according to their color. White [*PSI*^+^] colonies were subsequently grown overnight at 30°C and then replica plated onto ¼ YEPD and ¼ YEPD with 3 mM GdnHCl to confirm their [*PSI*^+^] phenotype. Statistical significance was determined with a Student’s t test.

### Toxicity Studies

74D-694 [*PSI*^+^]_weak_ and [*PSI*^+^]_strong_ strains carrying either the p6442 or G58X variants or the control plasmid p6431 were grown overnight at 30°C in SD-Ura medium. The cells were then diluted to an OD_600_ of 0.05 in 1ml of the same media, in a 24-well microtiter plate, CuSO_4_ was added, and growth was monitored for 60 hr in a FLUOstar OPTIMA plate reader (BMG Labtech). Three sequential 1:10 dilutions were performed, and the cultures were spotted onto agar plates containing SD-Ura medium with different concentrations of CuSO_4_ up to 800 μM.

### Peptide NMR Spectroscopy and Structural Calculations

NMR experiments were conducted on a four-channel Varian UnityINOVA 600 MHz NMR spectrometer (see the Supplemental Experimental Procedures).

## Supplemental Information

Document S1. Supplemental Experimental Procedures, Figures S1–S5, and Tables S1–S3
