# Supplementary material for: Structural Definition Is Important for the Propagation of the Yeast [PSI+] Prion
Source: Mol Cell. 2013 Jun 6;50(5):675–85. doi: 10.1016/j.molcel.2013.05.010 (PMC3679450; doi:10.1016/j.molcel.2013.05.010)
Supplement: Document S1. Supplemental Experimental Procedures, Figures S1–S5, and Tables S1–S3 [file mmc1.pdf]

Molecular Cell, Volume *50*

Supplemental Information

Structural Definition Is Important for the Propagation of the Yeast [*PSI*$^{+}$] Prion

Ricardo Marchante, Michelle Rowe, Jo Zenthon, Mark J. Howard, and Mick F. Tuite

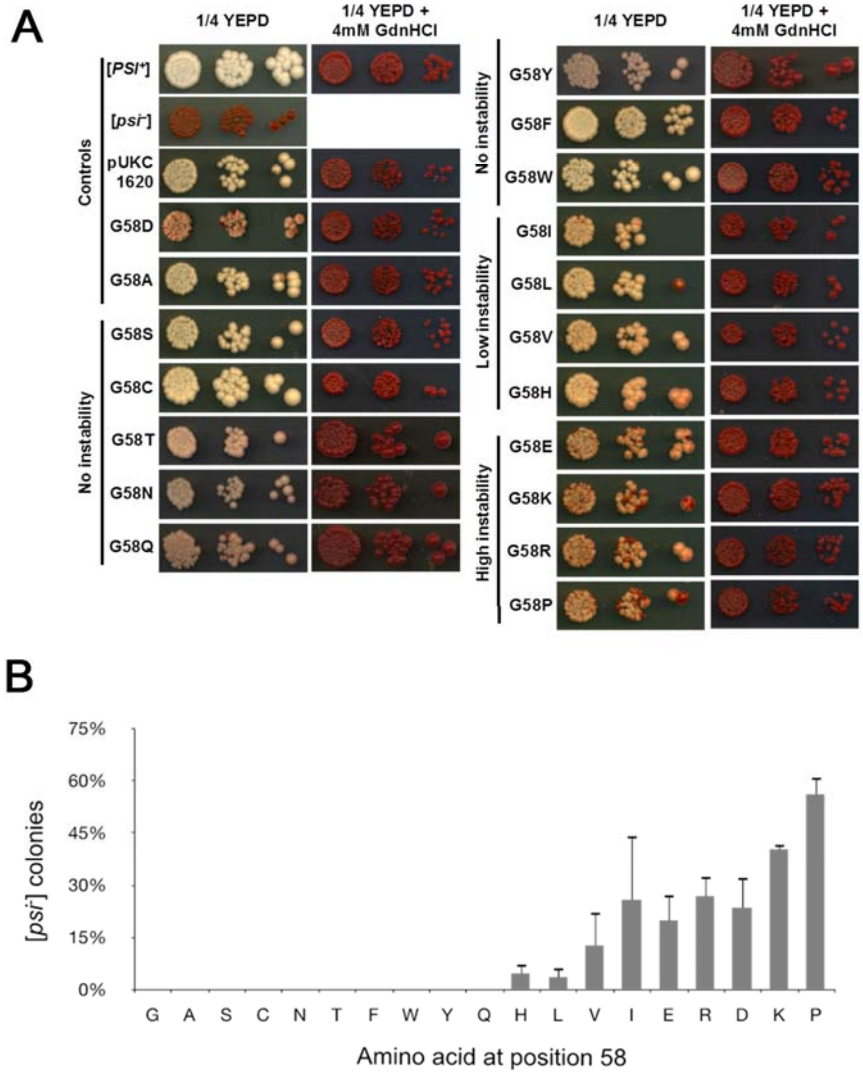

Figure S1. Phenotypic analysis of Sup35^{G58X} mutants, Related to Figure 1

A. G58 mutants show a relationship between the levels of instability and the biochemical properties of the amino acid introduced. G58 replacements with charged amino acids or Pro leads to high instability, whereas low instability is generally caused by hydrophobic amino acids. Small and aromatic amino acids show no phenotypic effect. **B.** Levels of instability caused by the different mutations were

assessed by plating single white/sectored colonies onto ¼ YEPD and scoring the number of red colonies that arise. Due to the stochasticity associated with the loss of [*PSI*$^+$], the percentages shown represent an average frequency at which the prion is loss and are usually associated with high standard deviations. The values represent the mean ± s.d. for three experiments.

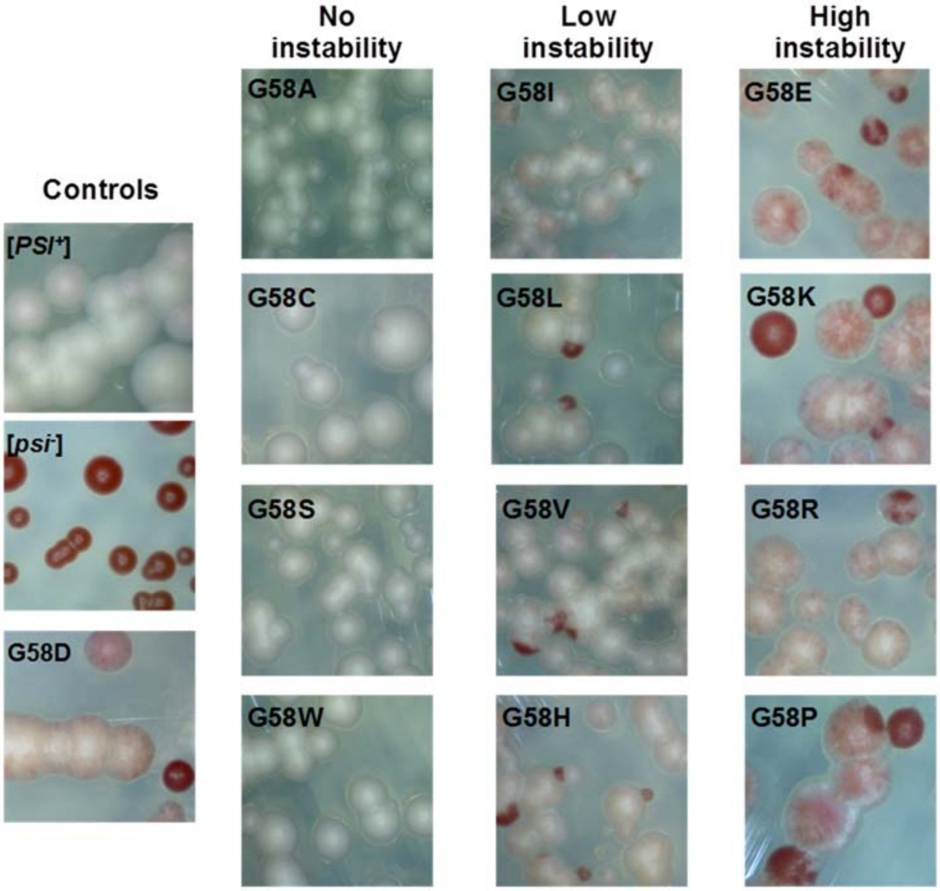

Figure S2. Phenotypic analysis of Sup35^{G58X} mutants - single colonies, Related to Figure 1

When observing magnified single colonies for each of the mutants, the instability phenotypes are exacerbated. Wild-type and mutants that cause no instability form smooth white colonies. Mutants causing low instability show light pink sectors in most colonies and produce [*psi*$^-$] colonies at a low frequency. Mutants that cause high instability show heavy red sectoring in all colonies and produce [*psi*$^-$] colonies at high frequency.

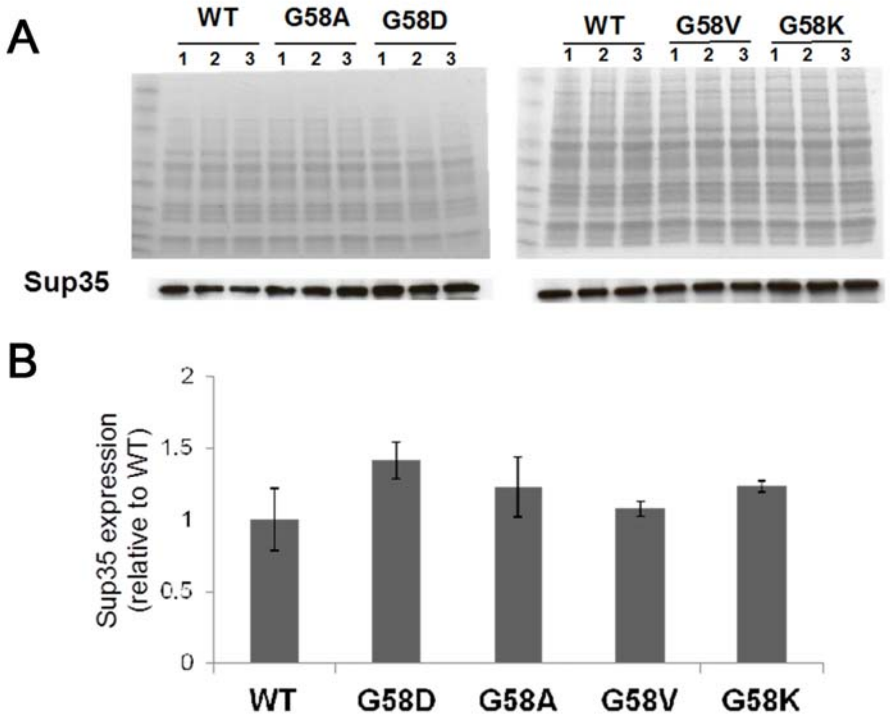

Figure S3. Western blot analysis of Sup35 levels in PNM2 mutants, Related to Figure 1

A. Western blot analysis demonstrates the [*PSI*$^{+}$] instability is a consequence of the introduced mutation rather than a consequence of a low level of expression of the mutant Sup35. Coomassie blue stained SDS-PAGE gels containing the same amount of total protein loaded used as a loading control. **B.** Relative levels of Sup35 expression, calculated using *ImageJ* software. The values represent the mean ± s.d. for three experiments.

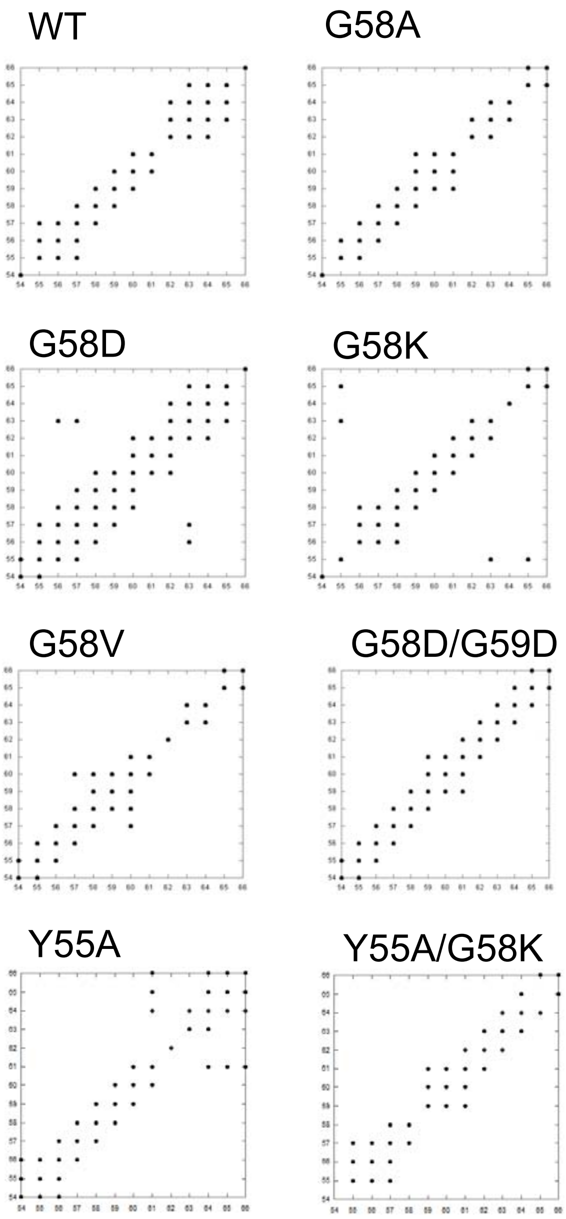

Figure S4. NOE/ROE contact maps for the structures calculated for Sup35 mutant peptides, Related to Figures 3 and 4

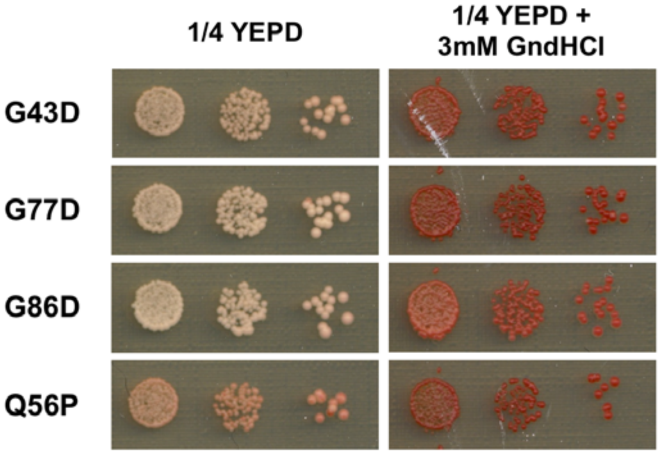

Figure S5. GdnHCl curing of [PSI$^+$] present in different oligopeptide repeat mutations, Related to Figure 5

G to D mutations in Sup35 oligopeptide repeats 1, 4 and 5 do not lead to [*PSI*$^+$] instability, while mutating the first Q in the second repeat to the canonical P present in other repeat does, highlighting the role of oligopeptide repeat 2 in prion propagation. The [*PSI*$^+$] phenotype in these mutants is readily reversible when they are grown in the presence of the prion curing agent GdnHCl.

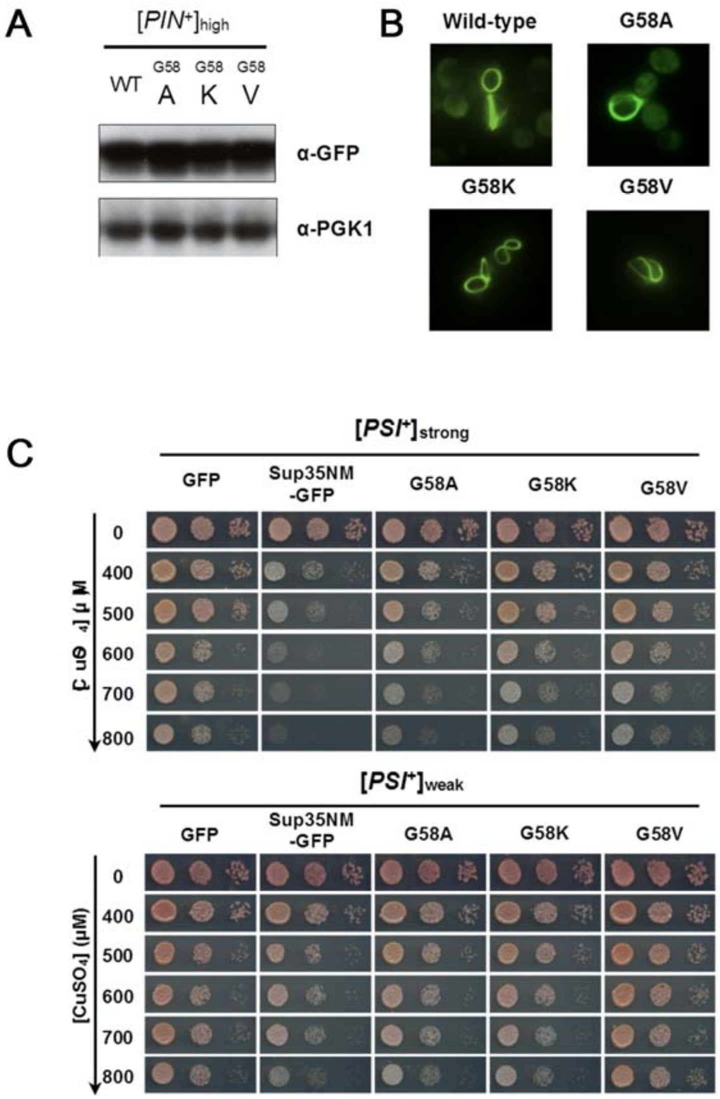

Figure S6. Mutant Sup35NM-GFP overexpression studies, Related to Figure 5

A. Western blot analysis shows overexpression of Sup35NM-GFP after a 5 hour induction course with 50μM $CuSO_4$. PGK1 was used as loading control **B.** After 4h overexpression of wild-type or mutant Sup35NM-GFP, in a wild-type [*psi*$^-$][*PIN*$^+$] background, ring structures characteristic of [*PSI*$^+$] induction are visible in some cells. **C.** Overexpression of Sup35NM-GFP elicits toxicity in a [*PSI*$^+$] background. The effects of overexpression first manifest as a whitening of the colonies (as cellular wild-type Sup35 is incorporated into aggregates and less available for translation termination), followed by cell death.

Table S1. NMR and refinement statistics for peptide structures, Related to Figures 3 and 4

	WT	G58D	G58A	G58K	G58V	G58D G59D	Y55A	Y55A G58K
**NMR distance and dihedral constraints**								
Distance constraints								
Total NOE	92	87	112	86	106	102	103	89
Intra-residue	56	48	73	47	59	50	62	50
Inter-residue	36	39	39	39	47	52	41	39
Sequential (|i-j|=1)	30	23	38	33	45	50	34	35
Medium-range (|i-j|<4)	6	13	1	1	2	2	6	4
Long-range (|i-j|>5)	0	3	0	5	0	0	1	0
**Structure Statistics**								
Violations (mean and s.d.)								
Distance constraints (Å)								
mean	0	0	0	0	0	0	0	0
s. d.	0.0004	0.0422	0.0130	0.0070	0.0080	0.0052	0.0064	0.0064
Max. distance constraint violation (Å)								
Deviations from idealized geometry								
Bond lengths (Å)	0	0	0	0	0	0	0	0
Bond angles (□)	0	0	0	0	0	0	0	0
Impropers (□)	0	0	0	0	0	0	0	0
Average pairwise r.m.s. deviation ** (Å)								
Heavy	3.333	2.596	3.405	2.969	2.918	3.494	3.002	3.551
Backbone	1.612	1.322	1.706	1.234	1.310	1.659	1.505	1.660

** Pairwise r.m.s. deviation was calculated among 20 refined structures over residues 56-62.

Table S2. Oligonucleotide sequences used in this study, Related to the Experimental Procedures

Residue modification	Oligonucl. Name	Oligonucleotide sequence (5’ → 3’)*
**G58D**	RM_G58D	TTATTCTGGGTACCAACAAGATGGCTATCAACAGTACAATC
**G58A**	RM_G58A	TTCTGGGTACCAACAAGCTGGCTATCAACAGTACA
**G58C**	RM_G58C	TCTGGGTACCAACAATGTGGCTATCAACAGTAC
**G58W**	RM_G58W	CAAGGTTATTCTGGGTACCAACAATGGGGCTATCAACAGTACAAT
**G58V**	RM_G58V	TTATTCTGGGTACCAACAAGTTGGCTATCAACAGTACAATC
**G58I**	RM_G58I	AATTACCAAGGTTATTCTGGGTACCAACAAATTGGCTATCAACAGTAC
**G58K**	RM_G58K	CCAAGGTTATTCTGGGTACCAACAAAAGGGCTATCAACAGTACAATCC
**G58P**	RM_G58P	ACCAAGGTTATTCTGGGTACCAACAACCTGGCTATCAACAGT
**G58Y**	RM_G58Y	TCTGGGTACCAACAATACGGCTATCAACAGTAC
**G58F**	RM_G58F	AATTACCAAGGTTATTCTGGGTACCAACAATTTGGCTATCAACAGTAC
**G58Q**	RM_G58Q	CCAAGGTTATTCTGGGTACCAACAACAGGGCTATCAACAGTACAATCC
**G58T**	RM_G58T	ACCAAGGTTATTCTGGGTACCAACAAACTGGCTATCAACAGT
**G58N**	RM_G58N	AATTACCAAGGTTATTCTGGGTACCAACAAAATGGCTATCAACAGTAC
**Random G58**	RM_G58X	CCAAGGTTATTCTGGGTACCAACAANNNGGCTATCAACAGTACAATCC
**Deletion G58**	RM_deltaG58	GGTTATTCTGGGTACCAACAAGGCTATCAACAGTACA
**G59A**	RM_G59A	TGGGTACCAACAAGGTGCCTATCAACAGTACAATC
**Random G59**	RM_G59X	GTTATTCTGGGTACCAACAAGGTNNNTATCAACAGTACAATCCCGACG
**G58D/G59D**	RM_G58G59D	TTATTCTGGGTACCAACAAGATGACTATCAACAGTACAATCC
**Y60A**	RM_Y60A	GGGTACCAACAAGGTGGCGCTCAACAGTACAATCCCGA
**Y60G**	RM_Y60G	GGGTACCAACAAGGTGGCGGTCAACAGTACAATCCCGA
**Y60W**	RM_Y60W	GGTACCAACAAGGTGGCTGGCAACAGTACAATCCCGAC
**Y60D**	RM_Y60D	GGTACCAACAAGGTGGCGATCAACAGTACAATCCC
**Q56P**	RM_Q56P	AGGTTATTCTGGGTACCCACAAGGTGGCTATCAAC
**G43D**	RM_G43D	TCAAGCCCAACCTGCAGATGGGTACTACCAAAATT
**G77D**	RM_G77D	CAGCAACAGTATAATCCTCAAGATGGCTATCAACAG
**G86D**	RM_86D	CAACAGTACAATCCTCAAGACGGTTATCAGCAGCAA
**Y55A**	RM_Y55A_G	ATTACCAAGGTTATTCTGGGGCCCAACAAGGTGGCTATCAAC
**Y55A + G58K**	RM_Y55A_K	CCAAAATTACCAAGGTTATTCTGGGGCCCAACAAAAGGGCTATCAACA GTACAATCCCGACG

* - ‘N’ designates a random nucleotide

Table S3. Plasmids used in this study, Related to the Experimental Procedures

Plasmid name	Plasmid description
pUKC1620	pRS313-P_{35}Sup35
pUKC1620-G58D	pRS313-P_{35}Sup35 (G58D)
pUKC1620-G58A	pRS313-P_{35}Sup35 (G58A)
pUKC1620-G58S	pRS313-P_{35}Sup35 (G58S)
pUKC1620-G58W	pRS313-P_{35}Sup35 (G58W)
pUKC1620-G58C	pRS313-P_{35}Sup35 (G58C)
pUKC1620-G58I	pRS313-P_{35}Sup35 (G58I)
pUKC1620-G58L	pRS313-P_{35}Sup35 (G58L)
pUKC1620-G58V	pRS313-P_{35}Sup35 (G58V)
pUKC1620-G58H	pRS313-P_{35}Sup35 (G58H)
pUKC1620-G58E	pRS313-P_{35}Sup35 (G58E)
pUKC1620-G58K	pRS313-P_{35}Sup35 (G58K)
pUKC1620-G58R	pRS313-P_{35}Sup35 (G58R)
pUKC1620-G58P	pRS313-P_{35}Sup35 (G58P)
pUKC1620-G58T	pRS313-P_{35}Sup35 (G58T)
pUKC1620-G58Q	pRS313-P_{35}Sup35 (G58Q)
pUKC1620-G58F	pRS313-P_{35}Sup35 (G58F)
pUKC1620-G58Y	pRS313-P_{35}Sup35 (G58Y)
pUKC1620-G58N	pRS313-P_{35}Sup35 (G58N)
pUKC1620-Δ58	pRS313-P_{35}Sup35 (Δ58)
pUKC1620-Y55A	pRS313-P_{35}Sup35 (Y55A)
pUKC1620-Y55A/G58K	pRS313-P_{35}Sup35 (Y55A/G58K)
pUKC1620-G5859D	pRS313-P_{35}Sup35 (G58D/G59D)
pUKC1620-G59A	pRS313-P_{35}Sup35 (G59A)
pUKC1620-G59L	pRS313-P_{35}Sup35 (G59L)
pUKC1620-G59R	pRS313-P_{35}Sup35 (G59R)
pUKC1620-Y60A	pRS313-P_{35}Sup35 (Y60A)
pUKC1620-Y60D	pRS313-P_{35}Sup35 (Y60D)
pUKC1620-Y60G	pRS313-P_{35}Sup35 (Y60G)
pUKC1620-Y60W	pRS313-P_{35}Sup35 (Y60W)

Table S3 (cont.)

Plasmid name	Plasmid description
pUKC1620-G43D	pRS313-P_{35}Sup35 (G43D)
pUKC1620-G77D	pRS313-P_{35}Sup35 (G77D)
pUKC1620-G86D	pRS313-P_{35}Sup35 (G86D)
pUKC1620-Q56P	pRS313-P_{35}Sup35 (Q56P)
p6442	P_{CUP1}Sup35NM-GFP
p6442-G58A	P_{CUP1}Sup35NM(G58A)-GFP
p6442-G58K	P_{CUP1}Sup35NM(G58K)-GFP
p6442-G58V	P_{CUP1}Sup35NM(G58V)-GFP
p6442-G5859D	P_{CUP1}Sup35NM(G58D/G59D)-GFP

Supplemental Experimental Procedures

Peptide NMR spectroscopy and structural calculations.

NMR experiments were recorded at 10°C on a four channel Varian UnityINOVA 600 MHz NMR spectrometer with a room temperature 5 mm HCN z-pulse field gradient triple resonance probe or a four channel Bruker Avance III 600 MHz NMR spectrometer with 5 mm QCI-cryoprobe using 1mM peptide samples in phosphate-buffered saline. 1H chemical shift referencing was based on the position of the water resonance with the exact value being related to the known relationship of the 1H2O resonance with temperature (Wishart and Sykes, 1994). For each sample, a 2D nuclear Overhauser effect spectroscopy (NOESY), rotational Overhauser effect spectroscopy (ROESY) and total correlation spectroscopy (TOCSY) experiment was recorded with mixing times of 300 ms, 150 ms and 80 ms respectively with acquisition times of 64 and 341 ms in the indirectly and directly acquired. In all experiments water suppression was obtained using WATERGATE based water suppression (Piotto et al., 1992). Data processing and analysis were undertaken using NMRPipe (Delaglio et al., 1995) and CCPN-Analysis (Fogh et al., 2002; Vranken et al., 2005). All peptide structural calculations were obtained using the Crystallography and NMR System (CNS) version 1.1 (Brunger et al., 1998) using all NOE/ROE contacts in one wide classification between 1.8-5.0 Å (Figure S4). Final structures were calculated from extended coordinates using the standard CNS NMR anneal protocol with sum averaging for dynamic annealing with NOEs from extended precursors (Brunger et al., 1998). A final structural ensemble of 20 structures for each sample was produced from which r.m.s. deviation values were obtained using MOLMOL version 2k.2 (Koradi et al., 1996). NMR and refinement statistics for the peptides are presented in Table S3.

Obtaining structural NMR data from peptides in the absence of any stabilizing solvent would be expected to produce a low number of significant NOE and ROE contacts to differentiate each peptide. Crucially we avoided stabilisers that could induce incorrect structural arrangements in each peptide. Observing nuclear Overhauser and rotating Overhauser contacts ensured rigorous and complete structural analysis and the experiments were tailored to provide optimum structural information through build-up analysis. To ensure non-bias, all ensembles described structurally and statistically were for the entire calculated set and not a sub-set of low energy calculated conformers.

Supplemental References

Brunger, A.T., Adams, P.D., Clore, G.M., DeLano, W.L., Gros, P., Grosse-Kunstleve, R.W., Jiang, J.S., Kuszewski, J., Nilges, M., Pannu, N.S., et al. (1998). Crystallography & NMR system: A new software suite for macromolecular structure determination. Acta Crystallogr D Biol Crystallogr 54, 905-921.

Delaglio, F., Grzesiek, S., Vuister, G.W., Zhu, G., Pfeifer, J., and Bax, A. (1995). NMRPipe: a multidimensional spectral processing system based on UNIX pipes. J Biomol NMR 6, 277-293.

Fogh, R., Ionides, J., Ulrich, E., Boucher, W., Vranken, W., Linge, J.P., Habeck, M., Rieping, W., Bhat, T.N., Westbrook, J., et al. (2002). The CCPN project: an interim report on a data model for the NMR community. Nat Struct Biol 9, 416-418.

Koradi, R., Billeter, M., and Wuthrich, K. (1996). MOLMOL: a program for display and analysis of macromolecular structures. J Mol Graph 14, 51-55, 29-32.

Piotto, M., Saudek, V., and Sklenar, V. (1992). Gradient-tailored excitation for single- quantum NMR spectroscopy of aqueous solutions. J Biomol NMR 2, 661-665.

Vranken, W.F., Boucher, W., Stevens, T.J., Fogh, R.H., Pajon, A., Llinas, M., Ulrich, E.L., Markley, J.L., Ionides, J., and Laue, E.D. (2005). The CCPN data model for NMR spectroscopy: development of a software pipeline. Proteins 59, 687-696.

Wishart, D.S., and Sykes, B.D. (1994). The 13C chemical-shift index: a simple method for the identification of protein secondary structure using 13C chemical-shift data. J Biomol NMR 4, 171-180.
